# 2-(3,3,4,4-Tetra­fluoro­pyrrolidin-1-yl)aniline

**DOI:** 10.1107/S1600536811030339

**Published:** 2011-08-02

**Authors:** Wanwan Cao, Jun-wen Zhong, Jin Wang, Pei-lian Liu, Zhuo Zeng

**Affiliations:** a School of Chemistry and Environment, South China Normal University, Guangzhou 510006, People’s Republic of China; b Key Laboratory of Organofluorine Chemistry, Shanghai Institute of Organic Chemistry, Chinese Academy of Sciences, Shanghai, 200032, People’s Republic of China

## Abstract

In the title fluorinated pyrrolidine derivative, C_10_H_10_F_4_N_2_, the dihedral angle between the best planes of the benzene and pyrrolidine rings is 62.6 (1)°. The crystal packing features inter­molecular N—H⋯F hydrogen bonds.

## Related literature

For applications of fluorinated pyrrolidine derivatives, see: Hulin *et al.* (2005 ▶); Kerekes *et al.* (2011 ▶); Marson (2005 ▶); Santora *et al.* (2008 ▶).
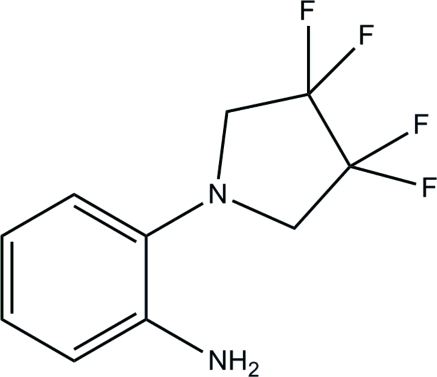


## Experimental

### 

#### Crystal data

C_10_H_10_F_4_N_2_
*M* *_r_* = 234.20
Orthorhombic, 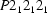

*a* = 6.791 (13) Å
*b* = 8.185 (16) Å
*c* = 18.66 (4) Å
*V* = 1037 (3) Å^3^
*Z* = 4
Mo *K*α radiation
μ = 0.14 mm^−1^
*T* = 298 K
0.30 × 0.28 × 0.22 mm

#### Data collection

Bruker SMART APEXII CCD area-detector diffractometer
Absorption correction: multi-scan (*SADABS*; Sheldrick, 1996 ▶) *T*_min_ = 0.959, *T*_max_ = 0.970
6022 measured reflections
1342 independent reflections
748 reflections with *I* > 2σ(*I*)
*R*_int_ = 0.061

#### Refinement

*R*[*F* ^2^ > 2σ(*F* ^2^)] = 0.041
*wR*(*F* ^2^) = 0.112
*S* = 1.02
1342 reflections
153 parameters
H atoms treated by a mixture of independent and constrained refinement
Δρ_max_ = 0.14 e Å^−3^
Δρ_min_ = −0.15 e Å^−3^

### 

Data collection: *APEX2* (Bruker, 2008 ▶); cell refinement: *SAINT* (Bruker, 2008 ▶); data reduction: *SAINT*; program(s) used to solve structure: *SHELXS97* (Sheldrick, 2008 ▶); program(s) used to refine structure: *SHELXL97* (Sheldrick, 2008 ▶); molecular graphics: *SHELXTL* (Sheldrick, 2008 ▶); software used to prepare material for publication: *SHELXL97*.

## Supplementary Material

Crystal structure: contains datablock(s) global, I. DOI: 10.1107/S1600536811030339/ld2018sup1.cif

Structure factors: contains datablock(s) I. DOI: 10.1107/S1600536811030339/ld2018Isup2.hkl

Supplementary material file. DOI: 10.1107/S1600536811030339/ld2018Isup3.cml

Additional supplementary materials:  crystallographic information; 3D view; checkCIF report

## Figures and Tables

**Table 1:** Hydrogen-bond geometry (Å, °)

*D*—H⋯*A*	*D*—H	H⋯*A*	*D*⋯*A*	*D*—H⋯*A*
N2—H2*A*⋯F1^i^	0.84 (5)	2.59 (5)	3.295 (8)	142 (4)

Symmetry code: (i) 
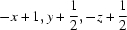
.

**Fig. 1.**
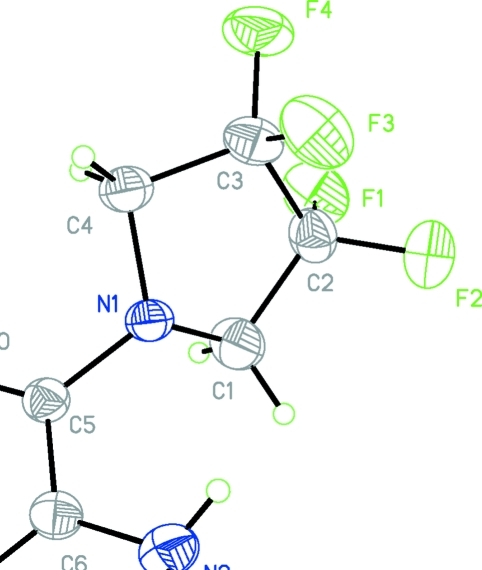
View of the title compound showing the atom-labelling scheme. Ellipsoids are drawn at the 50% probability level.

**Fig. 2.**
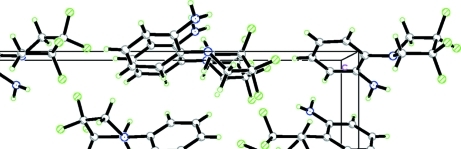
Perspective view of the crystal packing.

## supplementary crystallographic information

### Comment

Fluorinated pyrrolidine derivatives have attracted much attention due to their potential applications as dipeptidyl peptidase IV inhibitors (Hulin *et al.*, 2005), asymmetric synthesis catalysts (Marson, 2005), aurora kinase inhibitors (Kerekes *et al.*, 2011), and H3 receptor antagonists (Santora *et al.*, 2008). Herein, we report the crystal structure of the title compound (Fig. 1), obtained by the reaction of *o*-phenylenediamine with trifluoromethanesulfonic acid 2,2,3,3-tretrafluoro-1,4-butanediyl ester.

The dihedral angle between the plane of phenyl ring and the least-squares plane of pyrrolidine ring is 62.63 (14)°. The pyrrolidine ring adopts a distorted N1-envelope conformation with folding angle 40.6 (2)°. The crystal packing (Fig. 2) is characterized by intermolecular N—H···F—C bonds linking molecules in zigzag chains along b.

### Experimental

A mixture of trifluoromethanesulfonic acid 2,2,3,3,-tretrafluoro-1,4-butanediyl ester(1 mmol), *o*-phenylenediamine (1.5 mmol), Et_3_N (3 mmol) and ethanol(15 ml) was placed in a round-bottomed flask fitted with a reflux condenser, and then heated at reflux for 30 h. After cooling, the organic solvent was removed under reduced pressure and to the residue was solved in dichloromethane then washed with water, and the organic layer was dried over anhydrous Na_2_SO_4_. After the solvent was removed, the residue was purified by flash chromatography on silica gel to afford a purple solid (164 mg), yield 70%. Crystals suitable for X-ray structural analysis were grown from CH_3_CN solution at room temperature.

### Refinement

H-atoms were placed in calculated positions with C—H = 0.93 Å; the H atoms of amino group were refined freely.

Since this is a light-atom structure (it does not contain any atoms heavier than F) and since the data collection was carried out using Mo radiation, it was not possible to unambiguously determine the absolute configuration of this molecule. In the absence of significant anomalous scattering effects, Friedel pairs have been merged.

### Crystal data

**Table:**

C_10_H_10_F_4_N_2_	*F*(000) = 480
*M**_r_* = 234.20	*D*_x_ = 1.500 Mg m^−^^3^
Orthorhombic, *P*2_1_2_1_2_1_	Mo *K*α radiation, λ = 0.71073 Å
*a* = 6.791 (13) Å	µ = 0.14 mm^−^^1^
*b* = 8.185 (16) Å	*T* = 298 K
*c* = 18.66 (4) Å	Block, purple
*V* = 1037 (3) Å^3^	0.30 × 0.28 × 0.22 mm
*Z* = 4	

### Data collection

**Table:**

Bruker SMART APEXII CCD area-detector diffractometer	1342 independent reflections
Radiation source: fine-focus sealed tube	748 reflections with *I* > 2σ(*I*)
graphite	*R*_int_ = 0.061
φ and ω scans	θ_max_ = 27.0°, θ_min_ = 2.2°
Absorption correction: multi-scan (*SADABS*; Sheldrick, 1996)	*h* = −8→7
*T*_min_ = 0.959, *T*_max_ = 0.970	*k* = −8→10
6022 measured reflections	*l* = −23→23

### Refinement

**Table:**

Refinement on *F*^2^	Primary atom site location: structure-invariant direct methods
Least-squares matrix: full	Secondary atom site location: difference Fourier map
*R*[*F*^2^ > 2σ(*F*^2^)] = 0.041	Hydrogen site location: inferred from neighbouring sites
*wR*(*F*^2^) = 0.112	H atoms treated by a mixture of independent and constrained refinement
*S* = 1.02	*w* = 1/[σ^2^(*F*_o_^2^) + (0.038*P*)^2^ + 0.0935*P*] where *P* = (*F*_o_^2^ + 2*F*_c_^2^)/3
1342 reflections	(Δ/σ)_max_ < 0.001
153 parameters	Δρ_max_ = 0.14 e Å^−^^3^
0 restraints	Δρ_min_ = −0.15 e Å^−^^3^

### Special details

**Table:**

Geometry. All e.s.d.'s (except the e.s.d. in the dihedral angle between two l.s. planes) are estimated using the full covariance matrix. The cell e.s.d.'s are taken into account individually in the estimation of e.s.d.'s in distances, angles and torsion angles; correlations between e.s.d.'s in cell parameters are only used when they are defined by crystal symmetry. An approximate (isotropic) treatment of cell e.s.d.'s is used for estimating e.s.d.'s involving l.s. planes.
Refinement. Refinement of *F*^2^ against ALL reflections. The weighted *R*-factor *wR* and goodness of fit *S* are based on *F*^2^, conventional *R*-factors *R* are based on *F*, with *F* set to zero for negative *F*^2^. The threshold expression of *F*^2^ > σ(*F*^2^) is used only for calculating *R*-factors(gt) *etc*. and is not relevant to the choice of reflections for refinement. *R*-factors based on *F*^2^ are statistically about twice as large as those based on *F*, and *R*- factors based on ALL data will be even larger. Since this is a light atom structure (does not contain any atoms heavier than Si) and since the data collection was carried out using Mo radiation, it is not possible to unambiguously determine the absolute configuration of this molecule.

### Fractional atomic coordinates and isotropic or equivalent isotropic displacement parameters (Å^2^)

**Table:**

	*x*	*y*	*z*	*U*_iso_*/*U*_eq_	
F2	0.5502 (4)	0.9533 (4)	0.28755 (12)	0.0946 (9)	
F4	0.9856 (4)	0.9465 (4)	0.22927 (13)	0.0938 (9)	
F3	0.7878 (4)	1.1542 (3)	0.23004 (13)	0.0903 (9)	
F1	0.7058 (4)	0.7458 (3)	0.24699 (12)	0.0897 (8)	
C5	0.5124 (5)	0.9963 (4)	0.04139 (17)	0.0451 (8)	
C6	0.3393 (5)	1.0854 (4)	0.02680 (18)	0.0491 (9)	
C3	0.8125 (6)	1.0019 (5)	0.2037 (2)	0.0608 (10)	
C2	0.6375 (6)	0.8962 (5)	0.22754 (19)	0.0595 (11)	
C10	0.6062 (6)	0.9131 (5)	−0.01323 (19)	0.0575 (10)	
H10	0.7199	0.8541	−0.0031	0.069*	
C7	0.2663 (6)	1.0832 (5)	−0.0431 (2)	0.0605 (11)	
H7	0.1501	1.1383	−0.0536	0.073*	
C8	0.3635 (7)	1.0009 (5)	−0.0966 (2)	0.0688 (12)	
H8	0.3133	1.0027	−0.1430	0.083*	
C9	0.5346 (7)	0.9155 (5)	−0.0827 (2)	0.0716 (13)	
H9	0.6004	0.8608	−0.1192	0.086*	
C4	0.7993 (5)	0.9997 (5)	0.12353 (18)	0.0583 (10)	
H4A	0.8623	1.0946	0.1025	0.070*	
H4B	0.8569	0.9013	0.1036	0.070*	
C1	0.5016 (6)	0.8845 (5)	0.16406 (19)	0.0665 (12)	
H1A	0.5021	0.7752	0.1440	0.080*	
H1B	0.3679	0.9136	0.1771	0.080*	
N1	0.5854 (4)	1.0035 (4)	0.11341 (14)	0.0466 (7)	
N2	0.2447 (6)	1.1710 (5)	0.0805 (2)	0.0653 (10)	
H2A	0.312 (7)	1.201 (6)	0.116 (3)	0.091 (19)*	
H2B	0.171 (9)	1.254 (9)	0.068 (3)	0.17 (3)*	

### Atomic displacement parameters (Å^2^)

**Table:**

	*U*^11^	*U*^22^	*U*^33^	*U*^12^	*U*^13^	*U*^23^
F2	0.093 (2)	0.123 (2)	0.0670 (14)	0.0049 (17)	0.0166 (15)	−0.0149 (14)
F4	0.0610 (15)	0.127 (2)	0.0934 (19)	0.0073 (16)	−0.0274 (15)	0.0235 (16)
F3	0.118 (2)	0.0611 (15)	0.0915 (17)	−0.0124 (16)	−0.0215 (16)	−0.0148 (13)
F1	0.111 (2)	0.0665 (15)	0.0915 (17)	0.0003 (16)	−0.0187 (16)	0.0234 (14)
C5	0.041 (2)	0.047 (2)	0.0470 (18)	−0.0033 (19)	−0.0009 (17)	0.0050 (16)
C6	0.047 (2)	0.046 (2)	0.054 (2)	−0.0019 (18)	−0.0030 (19)	0.0006 (18)
C3	0.056 (3)	0.058 (3)	0.068 (2)	0.007 (2)	−0.016 (2)	0.004 (2)
C2	0.069 (3)	0.062 (3)	0.048 (2)	0.006 (2)	−0.002 (2)	0.008 (2)
C10	0.058 (2)	0.055 (2)	0.059 (2)	0.004 (2)	0.003 (2)	−0.0032 (19)
C7	0.057 (3)	0.061 (2)	0.064 (2)	0.000 (2)	−0.016 (2)	0.009 (2)
C8	0.090 (3)	0.069 (3)	0.047 (2)	−0.013 (3)	−0.013 (2)	0.000 (2)
C9	0.087 (4)	0.069 (3)	0.058 (3)	−0.005 (3)	0.005 (2)	−0.011 (2)
C4	0.044 (2)	0.070 (3)	0.061 (2)	−0.004 (2)	−0.0026 (19)	0.010 (2)
C1	0.064 (3)	0.074 (3)	0.061 (2)	−0.017 (2)	−0.005 (2)	0.017 (2)
N1	0.0369 (16)	0.0548 (18)	0.0480 (16)	−0.0026 (15)	−0.0024 (14)	0.0073 (15)
N2	0.052 (2)	0.073 (2)	0.071 (2)	0.011 (2)	0.001 (2)	−0.004 (2)

### Geometric parameters (Å, °)

**Table:**

F2—C2	1.350 (5)	C7—C8	1.374 (6)
F4—C3	1.347 (5)	C7—H7	0.9300
F3—C3	1.350 (5)	C8—C9	1.380 (6)
F1—C2	1.365 (5)	C8—H8	0.9300
C5—C10	1.382 (5)	C9—H9	0.9300
C5—C6	1.409 (5)	C4—N1	1.465 (5)
C5—N1	1.434 (5)	C4—H4A	0.9700
C6—N2	1.381 (5)	C4—H4B	0.9700
C6—C7	1.395 (5)	C1—N1	1.472 (5)
C3—C4	1.498 (6)	C1—H1A	0.9700
C3—C2	1.536 (6)	C1—H1B	0.9700
C2—C1	1.505 (6)	N2—H2A	0.84 (5)
C10—C9	1.384 (6)	N2—H2B	0.87 (7)
C10—H10	0.9300		
			
C10—C5—C6	119.8 (3)	C7—C8—C9	121.1 (4)
C10—C5—N1	123.5 (3)	C7—C8—H8	119.5
C6—C5—N1	116.6 (3)	C9—C8—H8	119.5
N2—C6—C7	121.3 (4)	C8—C9—C10	118.6 (4)
N2—C6—C5	120.7 (3)	C8—C9—H9	120.7
C7—C6—C5	118.1 (3)	C10—C9—H9	120.7
F4—C3—F3	106.8 (3)	N1—C4—C3	100.8 (3)
F4—C3—C4	113.7 (3)	N1—C4—H4A	111.6
F3—C3—C4	111.6 (3)	C3—C4—H4A	111.6
F4—C3—C2	112.5 (3)	N1—C4—H4B	111.6
F3—C3—C2	108.6 (3)	C3—C4—H4B	111.6
C4—C3—C2	103.7 (3)	H4A—C4—H4B	109.4
F2—C2—F1	103.9 (3)	N1—C1—C2	103.1 (3)
F2—C2—C1	113.9 (4)	N1—C1—H1A	111.2
F1—C2—C1	111.1 (3)	C2—C1—H1A	111.2
F2—C2—C3	112.7 (4)	N1—C1—H1B	111.2
F1—C2—C3	108.8 (3)	C2—C1—H1B	111.2
C1—C2—C3	106.4 (3)	H1A—C1—H1B	109.1
C5—C10—C9	121.5 (4)	C5—N1—C4	117.6 (3)
C5—C10—H10	119.3	C5—N1—C1	116.2 (3)
C9—C10—H10	119.3	C4—N1—C1	106.7 (3)
C8—C7—C6	121.0 (4)	C6—N2—H2A	117 (3)
C8—C7—H7	119.5	C6—N2—H2B	118 (4)
C6—C7—H7	119.5	H2A—N2—H2B	107 (5)
			
C10—C5—C6—N2	−179.2 (4)	C6—C7—C8—C9	1.1 (6)
N1—C5—C6—N2	−1.6 (5)	C7—C8—C9—C10	0.6 (6)
C10—C5—C6—C7	1.3 (5)	C5—C10—C9—C8	−1.3 (6)
N1—C5—C6—C7	178.9 (3)	F4—C3—C4—N1	159.5 (3)
F4—C3—C2—F2	93.9 (4)	F3—C3—C4—N1	−79.6 (4)
F3—C3—C2—F2	−24.1 (4)	C2—C3—C4—N1	37.0 (4)
C4—C3—C2—F2	−142.8 (3)	F2—C2—C1—N1	115.4 (4)
F4—C3—C2—F1	−20.8 (4)	F1—C2—C1—N1	−127.7 (4)
F3—C3—C2—F1	−138.8 (3)	C3—C2—C1—N1	−9.4 (4)
C4—C3—C2—F1	102.5 (3)	C10—C5—N1—C4	31.8 (5)
F4—C3—C2—C1	−140.6 (4)	C6—C5—N1—C4	−145.8 (4)
F3—C3—C2—C1	101.4 (4)	C10—C5—N1—C1	−96.3 (4)
C4—C3—C2—C1	−17.3 (4)	C6—C5—N1—C1	86.2 (4)
C6—C5—C10—C9	0.3 (5)	C3—C4—N1—C5	−177.7 (3)
N1—C5—C10—C9	−177.1 (4)	C3—C4—N1—C1	−45.2 (4)
N2—C6—C7—C8	178.5 (4)	C2—C1—N1—C5	167.3 (3)
C5—C6—C7—C8	−2.0 (6)	C2—C1—N1—C4	34.1 (4)

### Hydrogen-bond geometry (Å, °)

**Table:**

*D*—H···*A*	*D*—H	H···*A*	*D*···*A*	*D*—H···*A*
N2—H2A···F1^i^	0.84 (5)	2.59 (5)	3.295 (8)	142 (4)

Symmetry codes: (i) −*x*+1, *y*+1/2, −*z*+1/2.
